# Targeted Delivery of Deoxycytidine Kinase to Her2-Positive Cells Enhances the Efficacy of the Nucleoside Analog Fludarabine

**DOI:** 10.1371/journal.pone.0157114

**Published:** 2016-06-09

**Authors:** Sujatha P. Koduvayur, Ying Su, Brian K. Kay, Arnon Lavie

**Affiliations:** 1 Department of Biological Sciences, University of Illinois at Chicago, Chicago, IL 60607, United States of America; 2 Department of Biochemistry and Molecular Genetics, University of Illinois at Chicago, Chicago, IL 60607, United States of America; 3 The Jesse Brown VA Medical Center, Chicago, IL, United States of America; University of Nebraska Medical Center, UNITED STATES

## Abstract

Cytotoxic drugs, such as nucleoside analogs and toxins, commonly suffer from off-target effects. One approach to mitigate this problem is to deliver the cytotoxic drug selectively to the intended site. While for toxins this can be achieved by conjugating the cell-killing moiety to a targeting moiety, it is not an option for nucleoside analogs, which rely on intracellular enzymes to convert them to their active triphosphorylated form. To overcome this limitation, and achieve site-targeted activation of nucleoside analogs, we fused the coding region of a prodrug-activating enzyme, deoxycytidine kinase (dCK), to affinity reagents that bind to the Her2 cell surface protein. We evaluated dCK fusions to an anti-Her2 affibody and Designed Ankyrin Repeat Protein (DARPin) for their ability to kill cancer cells by promoting the activation of the nucleoside analog fludarabine. Cell staining and flow cytometry experiments with three Her2 positive cancer cell lines (BT-474-JB, JIMT-1 and SK-OV-3) indicate dCK fusions binding and cellular internalization. In contrast, these reagents bind only weakly to the Her2 negative cell line, MCF-7. Cell proliferation assays indicate that SK-OV-3 and BT-474-JB cell lines exhibit significantly reduced proliferation rates when treated with targeting-module fused dCK and fludarabine, compared to fludarabine alone. These findings demonstrate that we have succeeded in delivering active dCK into the Her2-positive cells, thereby increasing the activation of fludarabine, which ultimately reduces the dose of nucleoside analog needed for cell killing. This strategy may help establish the therapeutic index required to differentiate between healthy tissues and cancer cells.

## Introduction

The “Holy Grail" in cancer therapy is a drug that eliminates cancer cells, while sparing normal cells. Whereas most current drugs are relatively efficient at killing cancer cells, discrimination with healthy cells is suboptimal, and, as a result, chemotherapeutic cancer treatments are fraught with dose-limiting, toxic side effects. For example, nucleoside analog drugs, one of the earliest cancer drugs, and still a mainstay of cancer treatment, are efficient in causing DNA damage [1,2]. Such drugs predominantly target cells that are in S-phase, and this is the basis for whatever discrimination they have between cancer and healthy cells. However, such selectivity based primarily on cell cycle is not sufficient for non-toxic treatment, since many healthy cells undergo cell division. To solve the cancer cell versus healthy cell discrimination challenge, newer “targeted” therapeutics have been developed.

Antibody-drug conjugates (ADCs) have been designed along the lines of Paul Ehrlich’s ‘magic bullet’ concept, as the definitive method of targeted therapy towards diseases, like cancer [3,4]. Since the concept was first introduced in 1980, there are currently about 30 ADCs approved for clinical trials [5], of which only two have been cleared for marketing [4,6]. This low acceptance rate illustrates the challenges inherent in generating ADCs that meet the criteria for effective therapeutics: specificity, low off-target toxicity, and drug potency. ADCs are made of three components: the antibody moiety that provides target-specificity, the drug, which is the effector component, and a linker that connects the two moieties. The principal reasons for poor ADC effectiveness are related to the ideal construction and combination of these three components. All ADCs currently in clinical trials and market contain an IgG as the targeting moiety, which in turn brings with it inherent drawbacks, including off-target toxicity, triggered by its Fc region, which causes Antibody-Dependent Cell-Mediated Cytotoxicity (ADCC) and Complement Dependent Cytotoxicity (CDC) [7], and poor retention and penetration into tumors, due to its large size [8,9]. Challenges in conjugation chemistry are also a contributing factor to ineffective or low-quality ADCs. The heterogeneity of generated ADC molecules, based on the number of drug molecules loaded onto the ADCs [10,11], often results in poor potency of the ADC [12] and the need to use drugs at higher concentrations than those regularly used in chemotherapy [13,14]. Moreover, special attention must be paid to the nature of the linker itself, so as to prevent enzymatic degradation in extracellular fluids [15] and the resulting increase in systemic toxicity [16].

**Fig 1:**
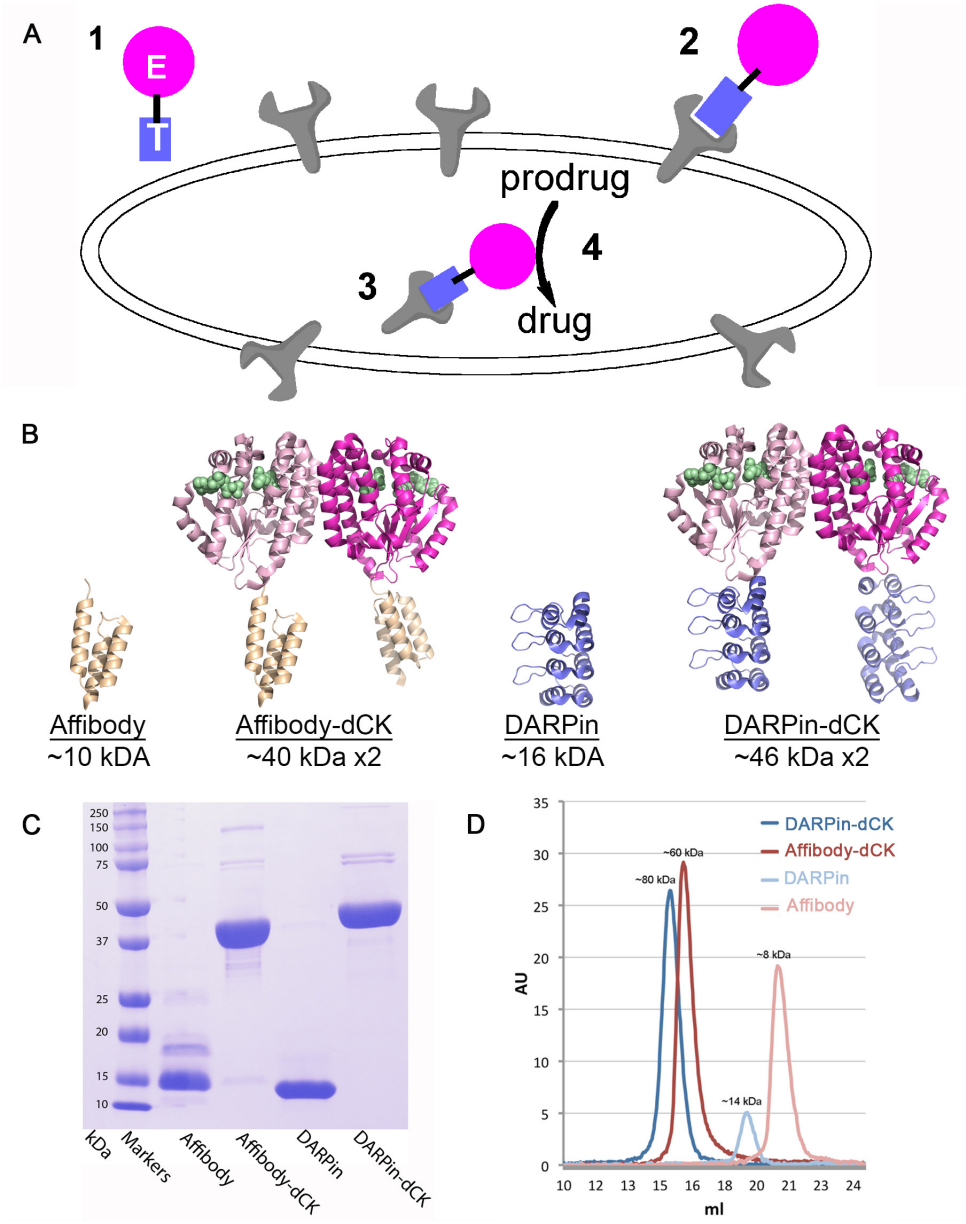
Strategy for preferential activation of prodrugs at target cells and the Her2-affinity reagents used in this study. (A) This strategy relies on a bi-modular fusion protein composed of a cell marker-targeting module (square labeled with T) genetically fused to an enzyme that catalyzes the activation of a prodrug (circle labeled with E). This fusion protein is administered systemically (point **1**), but it accumulates at the targeted cells by binding to a specific cell surface protein (point **2**). The fusion protein then enters the cell via receptor-mediated endocytosis or by membrane recycling (point **3**). Subsequent administration of an appropriate prodrug results in its preferential activation in the targeted cells (point **4**), thereby killing the targeted cell. (B) Ribbon diagram of the reagents with their molecular size indicated. Affibody, DARPin, and dCK models from PDB IDs 1LP1, 2JAB, and 1P5Z, respectively. The fusion proteins were modeled based on the individual structures. The dimeric nature of dCK result in molecules that contains two anti-Her2 modules, which are expected to increase its avidity to the receptor relative to the single affinity modules. Green spheres denote the substrates binding sites in dCK. (C) SDS-PAGE demonstrates the > 95% purity of the reagents. We note that the DARPin module runs as a smaller protein than the expected size. (D) Gel-filtration analysis of the reagents. The observed elution volumes correspond to the expected sizes of the reagents, with the fusion protein being dimeric, and the single affinity modules being monomeric.

We reasoned that an alternative bi-modular therapeutic approach, which combines a cancer cell selectivity module with a prodrug-activating module, could provide for preferential activation of the prodrug at the targeted cells (Fig 1A). It is the preferential activation of the prodrug at target cells that would provide a solution to the discrimination challenge between healthy and cancer cells. As a proof-of-concept, we designed a bi-modular system that combines a Her2-targeting module (Her2 is a receptor tyrosine protein kinase, also called erbB-2, Her2/neu), which has been shown to be over-expressed in certain breast cancers [17]), with a nucleoside-analog activating module. The Her2-targeting modules that we explored belong to two types of scaffolds: the designed ankyrin repeat protein (DARPin) [18] and the affibody [19]. For the nucleoside analog-activating module, we selected an engineered version of human deoxycytidine kinase (dCK) that has acquired enhanced enzymatic activity [20].

The DARPin and affibody scaffolds were chosen as these can be generated against specific targets by selecting for virtually any attribute that is present in an antibody (e.g., specificity, affinity, internalization, etc.) via in vitro selection methods like ribosome [21] or phage display [22]. Thus, any progress we make in this proof-of-concept work targeting Her2-positive cells could be expanded to other cancer cell markers. In addition, different to antibodies, these affinity scaffolds do not have Fc portions that trigger off-target effects, and their relatively small size leads to a greater diffusion rate into, and retention within, solid tumors [23,24]. While small proteins may have a short circulatory half-life, it remains to be determined, if, due to dimeric nature of our constructs which increases their size to above 40 kDa, the circulation time of constructs is sufficient for clinical efficacy.

Since we are coupling two protein molecules through genetic fusion, we are not restricted to chemical conjugation methods, but can use standard molecular cloning techniques to produce the affinity reagent-enzyme fusion proteins, thus ensuring a homogenous population of fusion proteins. Furthermore, fusion proteins may also impart low systemic toxicity, as they are less prone to degradation in extracellular fluids, compared to chemical linkers.

The nucleoside analog prodrug that we chose to use is fludarabine. A purine analog, fludarabine, is one of the most studied and extensively used in the treatment of B-cell chronic lymphocytic leukemia (B-CLL) [25]. These studies on fludarabine have shed light on the method of action of nucleoside analogs and its metabolism within the cell. The prodrug fludarabine is dephosphorylated before entering cells, where it is mono-phosphorylated by dCK, and subsequently di- and tri- phosphorylated [25,26,27,28]; the triphosphorylated form incorporates into DNA, resulting in cytotoxicity. dCK is the rate-limiting enzyme for the formation of fludarabine triphosphate [25,29]. Thus, delivery of our engineered dCK enzyme to the targeted cells should increase the amount of active fludarabine in the cells, thereby increasing its potency and cytotoxic specificity. Hence, this strategy may allow for nucleosides, which are not currently used for a breast cancer, to be incorporated into the treatment regimen.

Here, we show that we have successfully generated scaffold-dCK fusions targeted specifically for Her2 positive cancer cell lines. These fusion proteins are taken up preferentially by Her2 positive cell lines, compared to the Her2 negative cell lines, thereby indicating high specificity. Moreover, the internalized dCK enzyme is capable of enhancing the efficacy of the prodrug, fludarabine, as seen by decreased cell proliferation rates in Her2 positive cell lines treated with the scaffold-dCK reagent and the prodrug, compared to those treated with the prodrug alone. This work lays the groundwork for achieving increased selectivity between cancer and healthy cells, by enhancing the activation of nucleoside analog prodrugs at the targeted cells.

## Materials and Methods

### Cell lines and cell culture

The SK-OV-3 cell line was purchased (ATCC^®^ HTB-77^™^), the BT-474-JB and JIMT-1 cells lines were gifts of Dr. Maurizio Scaltriti (Memorial Sloan Kettering Cancer Center), and the MCF-7 cell line was a gift from Dr. Jack Kaplan (UIC). All cell lines were grown in Dulbecco's modified Eagle's medium (DMEM), with 10% fetal bovine serum, 2 mM L-glutamine, 50 units/mL penicillin G, and 50 μg/mL streptomycin, at 37°C with 5% CO_2_, according to standard cell culture techniques.

### Constructs, protein expression and purification

The coding regions for DARPins and affibodies, which bind the ectodomain of human Her2 [30], was synthesized by GenScript and a gift from Dr. Manfred Konrad, respectively. The genes were inserted into a modified version of the pET14b (Novagen), which contains the coding regions for (Small Ubiquitin-like Modifier) SUMO and human deoxycytidine kinase (dCK) [20,31]. To generate a version of the DARPin/affibody that is not conjugated to dCK enzyme, two stop codons were introduced between the coding regions of the two genes (i.e DARPin/affibody and dCK) by the QuikChange II kit (Agilent Technologies) according to the manufacturer’s instruction. To allow dye conjugation of the cysteine-free DARPin module, a cysteine residue was introduced at the C-terminus of DARPin coding region. Since dye conjugation of dCK-containing proteins resulted in reduced enzymatic activity, the cysteines at position 45 and 146 in the dCK were mutated to serines, which resulted in construct with no loss of catalytic activity upon dye labeling. The resulting constructs were used to transform BL21 DE bacterial cells (Novagen) for protein expression and purification.

Transformed bacterial cells were grown in 5 mL 2xYT media (per liter 16 g tryptone, 10 g yeast extract, 5 g NaCl), supplemented with 100 μg/mL ampicillin, overnight at 37°C. This starter culture was then used to inoculate 1L of 2xYT media, supplemented with 100 μg/mL ampicillin, and grown as before until an optical density (OD) of 0.6 was reached, at which time 0.2 mM isopropyl-β-D-thiogalactopyranoside (IPTG) was then added to the culture. After overnight shaking of the culture at 18°C and 190 rpm, the cells were pelleted, lysed in 50 mM Tris pH 7.5, 500 mM NaCl, 10% glycerol, 1% Triton X-100, and 1 mM phenylmethylsulfonyl fluoride (PMSF) for 30 min, and sonicated for 10 min (50% amplitude, 30 sec on/30 sec off pulse). Cell debris was pelleted at 33,000 rpm for 45 min at 4°C, and the supernatant was added to a pre-equilibrated 5 mL HisTrap^™^ HP FPLC column, at a flow rate of 2 mL/min. The column was washed with buffer (50 mM Tris-HCl pH 7.5, 500 mM NaCl, 25 mM imidazole) at 4 mL/min and proteins eluted with elution buffer (50 mM Tris-HCl, pH 7.5, 500 mM NaCl, 250 mM imidazole). The eluted protein was cleaved with in-house generated SUMO protease (1:200 weight:weight SUMO protease:protein) at room temperature for 2 h in 0.5 mM NaH_2_PO_4_ pH 7.5, 500 mM NaCl, 20 mM imidazole, 1 mM, Tris(2-carboxyethyl)phosphine (TCEP) (Hampton Research). The fully digested protein sample was then chromatographed on HisTrap^™^ resin, and the fusion proteins purified, as described above.

### AlexaFluor^®^647 labeling of proteins

Proteins were labeled with dye according to the manufacturer’s (Life Technologies) protocol. Briefly, 1 mg of protein was incubated with 10 μL of Alexa Fluor^®^ 647 (Life Technologies) in labeling buffer (50 mM Na_2_HPO_4_, 300 mM NaCl, 1 mM TCEP, pH 7.25) for 2 h at room temperature. Dye-labeled protein was purified by chromatography on a Superdex^™^200 10/300 GL (GE Healthcare Life Sciences) size-exclusion column.

### Enzyme linked immunosorbent assay (ELISA)

Binding of affinity module alone and of affinity-module-dCK fusion proteins to ectodomain IV of Her2 was detected by performing a protein ELISA as described before [32]. Briefly, 20 nM mouse anti-His antibody (Invitrogen) was used to capture 20 nM of recombinant human ErbB2/Her2 Fc Chimera (R&D Systems) onto Nunc polystyrene microtiter plate wells (Thermo Fisher Scientific). Fifty nM of the affinity module alone or of the affinity-module-dCK fusion protein were added to the wells, and binding detected with an anti-Myc antibody (9E10) conjugated to biotin (Santa Cruz Biotechnology) and streptavidin-conjugated to horseradish peroxidase (HRP; Abcam). The human Her2 ELISA Kit (Invitrogen) was used to determine the number of Her 2 receptors in cell lines. Briefly, 10^7^ cells were lysed in cell extraction buffer (Invitrogen), diluted to a volume of 100 μL/well, and the copy number of Her2 receptor/ cell calculated according to the manufacturer’s protocol.

### Cell staining

Cells were harvested by incubating with Trypsin-EDTA (Mediatech Inc.) at 37°C for 3–10 min. One hundred μL of cells (~ 2x10^4^ cells) was incubated with 5 μM of dye-labeled affibody or DARPin fusion protein and 1 μM of DARPin-dCK or 2 μM of affibody-dCK for 2 h at 4°C or 37°C. The cells were washed three times in wash buffer containing phosphate buffered saline (PBS; 137 mM NaCl, 3 mM KCl, 8 mM Na_2_HPO_4_, 1.5 mM KH_2_PO_4_), 1% fetal bovine serum (Hyclone), 0.1% sodium azide (Sigma), and resuspended in slide mounting buffer (wash buffer with 20% fetal bovine serum). The cells were then transferred onto glass slides by centrifuging them in a cytospin at 700 rpm for 5 min. Cells were incubated in 100 μL of 4,6-diamidino-2-phenylinodole (DAPI) for 10 min at room temperature, before adding mounting medium (Invitrogen). Cells were imaged in a Zeiss LSM5 confocal microscope and analyzed using software, according to the manufacturer’s protocol. For internalization assays, cells were grown in 24 well plates at 5x10^4^ cells/well in 150 μL, in the presence of 15 μL of CellLight^®^Early Endosomes-GFP, BacMam 2.0, CellLight^®^Late Endosomes-GFP, BacMam 2.0 and CellLight^®^Lysosome-GFP, BacMam 2.0 each (PPC of 30), overnight at 37°C. The cells were then washed and treated with dye-labeled protein (as above), before trypsinization and imaging.

### Flow cytometry

One hundred μL of 1x10^6^ cells were incubated with 1 μM of affinity reagents (DARPin and affibody, alone or fused to dCK) for 30 min at 4°C or 2 h at 37°C. Cells were washed three times with wash buffer, and clumps removed by passing the cells through a cell-strainer (Becton Dickinson). Cells were counted in a CyAn^™^ADP flow cytometer (Beckman Coulter), and data analyzed using Summit 4.3 software.

### Cell proliferation assay

Ninety-six well plates were seeded with 7x10^3^ cells/well and allowed to adhere overnight at 37°C in DMEM (Mediatech Inc.), 10% fetal bovine serum, 50 units/mL penicillin G, and 50 μg/mL streptomycin. Ten μL of 0.25 mM fludarabine des-phosphate (Sigma Aldrich), 5 μM DARPin/DARPin-dCK, 10 μM affibody/affibody-dCK or 10 μM dCK were added to the cells on days 0 and 2, and incubated at 37°C. Twenty μL of AlamarBlue^®^ reagent (Life Technologies Inc.) was added to cells and incubated at 37°C, 5% CO_2_ for 3.5 h before reading fluorescence intensity at 540–570 nm wavelength.

### Statistical Analysis

All quantitative data are analyzed statistically by measuring averages and standard deviations. Results of analysis are shown as error bars in corresponding graphs. For cell proliferation assay data, significance values (p values) were calculated using standard Student's T-Test one-tailed, type-2 analysis module in Microsoft excel.

## Results and Discussion

As a first step in creating an efficient anti-cancer modality, we generated several anti-Her2 module-engineered enzyme dCK fusion constructs (Fig 1B and S1 Fig). The two affinity modules we chose for this purpose were the anti-Her2 DARPin [30] and affibody [33], as they have been shown to bind tightly to the target ectodomain IV of Her2, with affinities of 90 pM and 200 pM, respectively. The dCK variant, which we chose to construct fusions to these two affinity reagents, contains two mutations that enhance its activity [20]. We overexpressed and purified the individual anti-Her2 modules alone (DARPin and affibody) and fused to dCK (i.e., DARPin-dCK, affibody-dCK), at high purity, as judged by SDS-PAGE (Fig 1C). The dimeric nature of dCK provides an added benefit to the system, by presenting two copies of the Her2-binding module. Gel filtration analysis confirmed that the fusion constructs migrate at a molecular size consistent with the dimeric species (Fig 1D). To enable easy detection in cell biology experiments, the purified proteins were labeled via maleimide chemistry, with Alexa Fluor^®^ 647 dye, at accessible cysteine residues.

**Fig 2:**
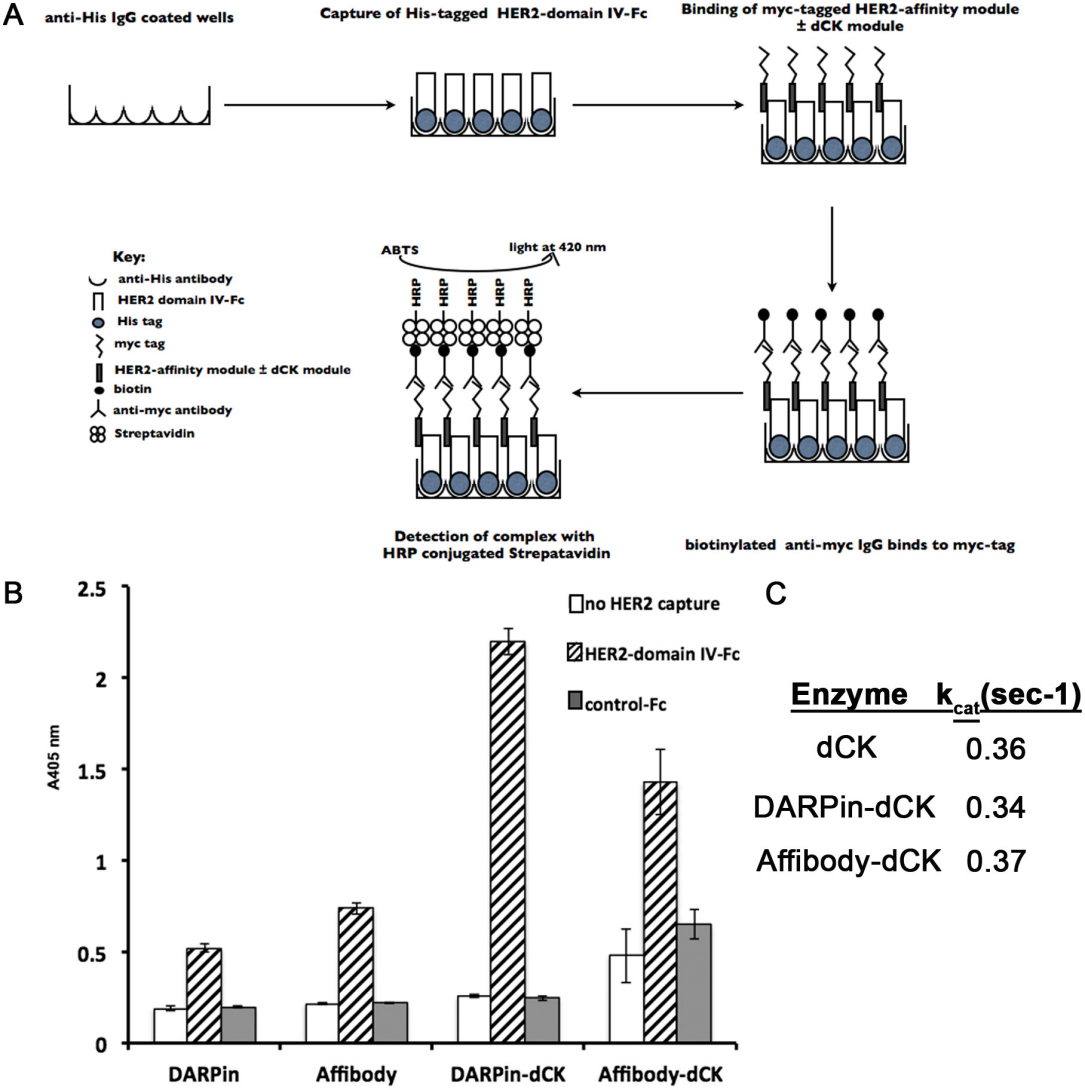
Characterizing individual anti-Her2 modules and the bi-modular anti-Her2-dCK fusion proteins. (A) Schematic of the ELISA assay used to evaluate the binding of the affinity reagents to the ectodomain IV of the Her2 receptor. (B) Binding of reagents to the ectodomain IV of Her2 receptor was measured by incubating 50 nM of reagents with 20 nM of immobilized Her2-Fc recombinant protein. Detection was performed using biotinylated anti-c-myc antibody and streptavidin conjugated to Horseradish peroxidase (HRP). Error bars represent standard deviation of triplicate measurements. We interpret the increased ELISA signal for the fusion proteins relative to the individual affinity modules resulting from the dimeric nature of the fusion proteins. (C) The observed steady state phosphorylation rate (k_obs_) of fludarabine by the engineered dCK (dCK-DMS74E) with, or without, the Her2-affinity modules is measured with 200 μM of fludarabine at 37°C. These measurements indicate that the affinity modules do not interfere with the dCK enzymatic activity.

Using an ELISA assay, we confirmed the binding of the above reagents to the ectodomain IV of Her2 [34]. The principles of the ELISA assay are schematically shown in Fig 2A. In the ELISA, the DARPin and affibody modules bind equally well to the Her2 domain IV (Fig 2B, left hashed bars). Notably, the anti-Her2 module-dCK fusion proteins (i.e., affibody-dCK or DARPin-dCK fusions) maintained their ability to bind Her2 (Fig 2B, right hashed bars), and in fact, showed an increased binding signal compared to the monomeric anti-Her2 modules alone. The apparent increase in the binding signal of the fusion proteins can be explained by avidity, as the dCK enzyme forms a dimer in solution (Fig 1D). As controls, we performed the ELISA omitting the Her2 capture step (white bars, Fig 2B) or by using a His-tagged Fc protein lacking the Her2 domain IV (gray bars, Fig 2B); in both cases the ELISA signal was very low, as expected. These controls demonstrate that we do not have non-specific binding of our Her2-affinity reagents to the ELISA plates or to the Fc portion that was present in the Her2 protein preparation.

After confirming the retained Her2-binding property of the anti-Her2 modules in the context of the fusion protein, we next checked the enzymatic activity of dCK. As anticipated, fusion of the Her2-specific modules to the dCK enzyme does not affect the kinase activity of dCK enzyme (Fig 2C). These results indicate that we were successful in generating fusion proteins that bind to Her2, and that the enzymatic activity of dCK in the context of the fusion construct is maintained.

### Bi-modular anti-Her2-dCK fusion proteins are capable of binding Her2 receptors in Her2-positive cancer cell lines

Having successfully generated enzymatically active DARPin- and affibody-dCK fusion proteins that bind to the ectodomain IV of Her2 *in vitro*, the next step was to assess if these fusion proteins can bind Her2-expressing cancer cells. An additional goal of these experiments was to assess the cellular localization of the anti-Her2 module-dCK proteins. Our ultimate goal of promoting the intracellular activation of nucleoside analog prodrugs requires the internalization of the fusion protein.

The ability to bind to cells expressing the Her2 receptor and the cellular localization was examined by confocal microscopy and by flow cytometry. The cell lines tested included two Her2-positive cancer cell lines, SK-OV-3 (ovarian cancer cell line [35]) and BT-474-JB (breast cancer cell line, [36]), and as the Her2-negative cell line, MCF-7 [37].

**Fig 3:**
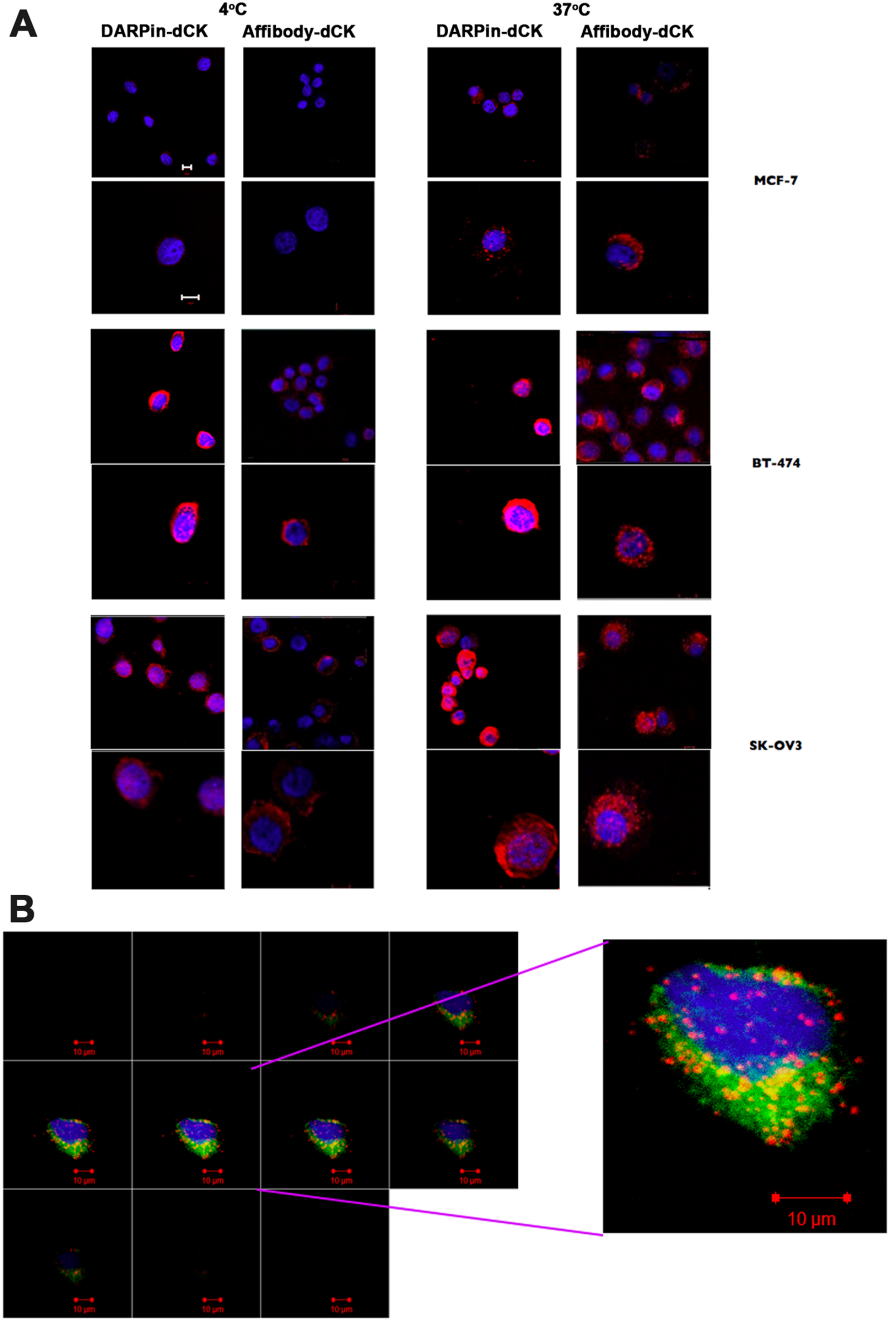
Binding of bi-modular anti-Her2 -dCK fusion proteins to cancer cells. (A) One hundred thousand cells were treated with 1 μM of Alexa Fluor^®^ 647 dye (red in the images) conjugated reagents and incubated for 2 h at either 4°C or 37°C. The nuclei of treated cells were stained with DAPI (blue) and visualized at 40x using a Zeiss confocal laser scanning microscope. Images are representative of three independent trials. Scale bar depicts 10 μm. Note the much-increased signal for the fusion proteins with the Her2-postive cells lines (BT-474 and SK-OV3), relative to the Her2-negative MCF-7 cell line. The stronger intracellular signal at 37°C relative to that observed at 4°C indicates that at least a fraction of the fusion proteins has internalized. (B) Internalization of DARPin-dCK in BT-474 cells. Co-localization of receptor bound reagents with intracellular vesicles was detected by treating 5x10^4^ cells with 1.5x10^6^ particles each of CellLight^®^ Reagents *BacMam 2.0* GFP markers for early and late endosomes and lysosomes, for 18 h at 37°C, according to the manufacturer’s protocol. These cells were then treated with 0.5 μM of Alexa Fluor^®^ 647 conjugated DARPin-dCK, as in A. Image sections at the z plane at 40x resolution of a single BT-474 cell are shown on the left panel. A high power image of a single section is shown on the right panel. GFP fluorescence indicates location of endosomes and lysosomes, while red fluorescence indicates the cellular location of DARPin-dCK. Image shown is representative of 10 cells imaged from duplicate trials. Scale bar is 10 μm.

Cells were incubated with DARPin-dCK and affibody-dCK proteins followed by confocal microscopy, which was conducted at both 4°C and 37°C since endocytosis is dependent on the higher temperature. Comparing the images taken at 4°C and 37°C would therefore allow us to assess the internalization of our constructs versus simply binding to the extracellular receptors. As expected, we see only weak staining of the Her2-negative MCF-7 cell line, suggesting little or no binding of the DARPin-dCK and affibody-dCK proteins (Fig 3A) to cells that do not express Her2. The images are significantly different with the Her2-positive cell lines (BT-474-JB and SK-OV-3) that show staining at both the 4°C and 37°C. Notably, at 37°C, we see a fluorescent signal that is more perinuclear, compared to surface staining, which we interpret as cell internalization of the fusion proteins.

To identify the cellular destination of DARPin-dCK further, we used fluorescently labeled markers of late endosomes and of lysosomes (see Methods), in addition to our Alexa Fluor^®^ 647 labeled DARPin-dCK (Fig 3B). The red signal from the Alexa dye conjugated DARPin-dCK and the green signal from the GFP markers appear only in sections that show blue staining of the nucleus coming from DAPI (middle sections) and not earlier or later (first and last sections), denoting internalization of the fusion proteins under these staining conditions. The co-localization of the GFP and Alexa signals (yellow, Fig 3B) indicates localization of the DARPin-dCK fusion protein within cytoplasmic organelles rather than being bound to the extracellular face of the cell membrane. The lack of continuous cytoplasmic signal for the DARPin-dCK protein suggests its location is predominately in endocytic vesicles. While this experiment shows that the fusion protein does become internalized, it is not clear how long it survives in the cell before being degraded in the lysosomes. Since we need active dCK within the cell to activate the nucleoside analog, a long cellular half-life for the dCK fusions would be highly advantageous. We investigate this issue below.

**Fig 4:**
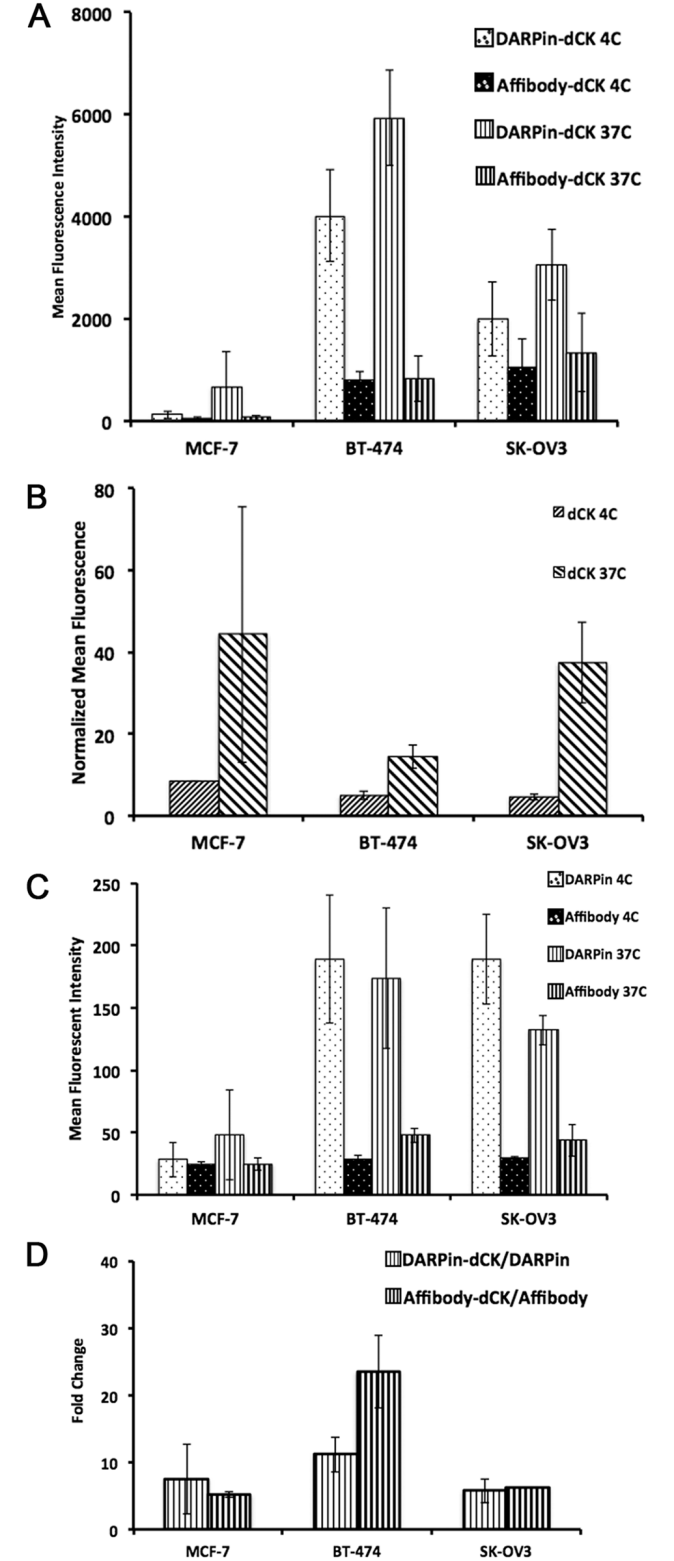
Binding of the bi-modular fusion proteins to cancer cells measured by flow cytometry. (A) One million cells were treated with 1 μM of reagents as in Fig 2 and mean fluorescence intensity was measured using a Cyan3 fluorescent cell sorter (Beckman). Fluorescent cells were gated on cells treated with reagents that were not conjugated to dye and hence did not exhibit any fluorescence at 647 nm. Error bars correspond to standard deviations on three independent trials. (B) One million cells were treated with 1 μM dCK-AlexaFluor^™^647 and the signal intensity was normalized to number of dye conjugations (ref 3 from Supplement). Error bars correspond to standard deviations of n = 3 trials. (C) Cell were treated with 2 μM of anti-Her2 DARPin-AlexaFluor^™^647 and Affibody-AlexaFluor^™^647 and measured as above. Note the much reduced mean fluorescence intensity in comparison to the fusion constructs (panel A). Error bars correspond to standard deviations of three independent trials. (D) Fluorescence intensities of cells treated with dCK-fusion protein were normalized to number of conjugated dye molecules (3), background subtracted and compared to those of cells treated with the Her2 affinity module (DARPin or affibody) alone. The resulting mean fold change in intensity was plotted for each cell type and reagent.

To confirm the confocal microscopy results, which indicate both specificity to Her2-positive cells and internalization of the fusion protein, we employed flow cytometry. A comparison of the Her2-positive BT-474-JB and SK-OV-3 cell lines to the Her2-negative MCF-7 cell line by flow cytometry demonstrated that the anti-Her2 module-dCK fusion protein shows a very weak signal with the MCF-7 cell line (Fig 4A) and a strong signal with the Her2-positive cell lines. Interestingly, with both Her2-positive cell lines we observe a stronger signal with the DARPin-dCK fusion protein compared to the affibody-dCK fusion protein. This could be a result of the difference in affinities, 90 pM *versus* 200 pM [30,33], between the two affinity modules to the Her2 receptor.

To verify that the flow signal is due to the interaction between the affinity module of the fusion protein and not to non-specific interactions with dCK, we repeated the experiment using dCK alone (i.e., dCK lacking the Her2 affinity module). The flow signal with dCK alone (Fig 4B) is > 2-orders of magnitude lower than the signal with the fusion protein (Fig 4A), demonstrating that it is indeed the Her2-affinity module that is responsible for the signal measured in the flow experiment.

Between the two Her2-positive cell lines, more Her2-affinity module-dCK fusion protein bound to BT-474-JB cells relative to SK-OV-3 cells (Fig 4A), despite SK-OV-3 cells having more Her2 receptors per cell (S2 Fig panel A). This observation could be a reflection of different receptor recycling dynamics in the two cell types and/or a difference in the distribution of the receptors on the cell surfaces. The stronger flow cytometry signal at 37°C versus 4°C is taken as indication of the internalization that occurs at the physiological temperature (Fig 4A).

Important to our overall strategy of delivering active dCK into Her2-positive cells, we find that fusing the enzyme to the anti-Her2 modules significantly enhances the flow signal relative to the anti-Her2 modules by themselves (Fig 4C and 4D). This observation suggests that the dimeric nature of the molecule, provided by dCK, plays an important role in enabling better cross-linking of receptors by the dimeric fusion-proteins than the monomeric anti-Her2 modules. Taken together, these cell culture results indicate that we were able to target the anti-Her2 module-dCK fusion proteins to Her2-positive cells and that some of the fusion protein internalizes.

### Combination treatment with anti-Her2 module-dCK fusion proteins and nucleoside analog drugs blocks cell proliferation more efficiently than treatment with either agent alone

The next step was to determine if these recombinant proteins, in combination with the nucleoside analog fludarabine, were effective at inhibiting the proliferation of Her2-positive cancer cell lines. To do this, we measured the effect on cell proliferation of these reagents singly (i.e., DARPin-dCK by itself, and fludarabine by itself), and in combination (i.e., DARPin-dCK plus fludarabine). Whereas DARPin-dCK by itself is relatively non-toxic to cells, a high concentration of fludarabine alone, due to endogenous expression of dCK, inhibits cells proliferation. Therefore, based on a fludarabine dose response with the examined cell lines (S3 Fig), we chose a concentration of the nucleoside analog that only marginally inhibits cell proliferation. We used the addition of dCK lacking the Her2-targeting module as an additional experimental control to demonstrate that dCK on its own is not toxic and that it is the internalization of dCK, made possible by the binding of the fusion constructs to the Her2 receptor, that contributes to the enhanced activation of fludarabine.

**Fig 5:**
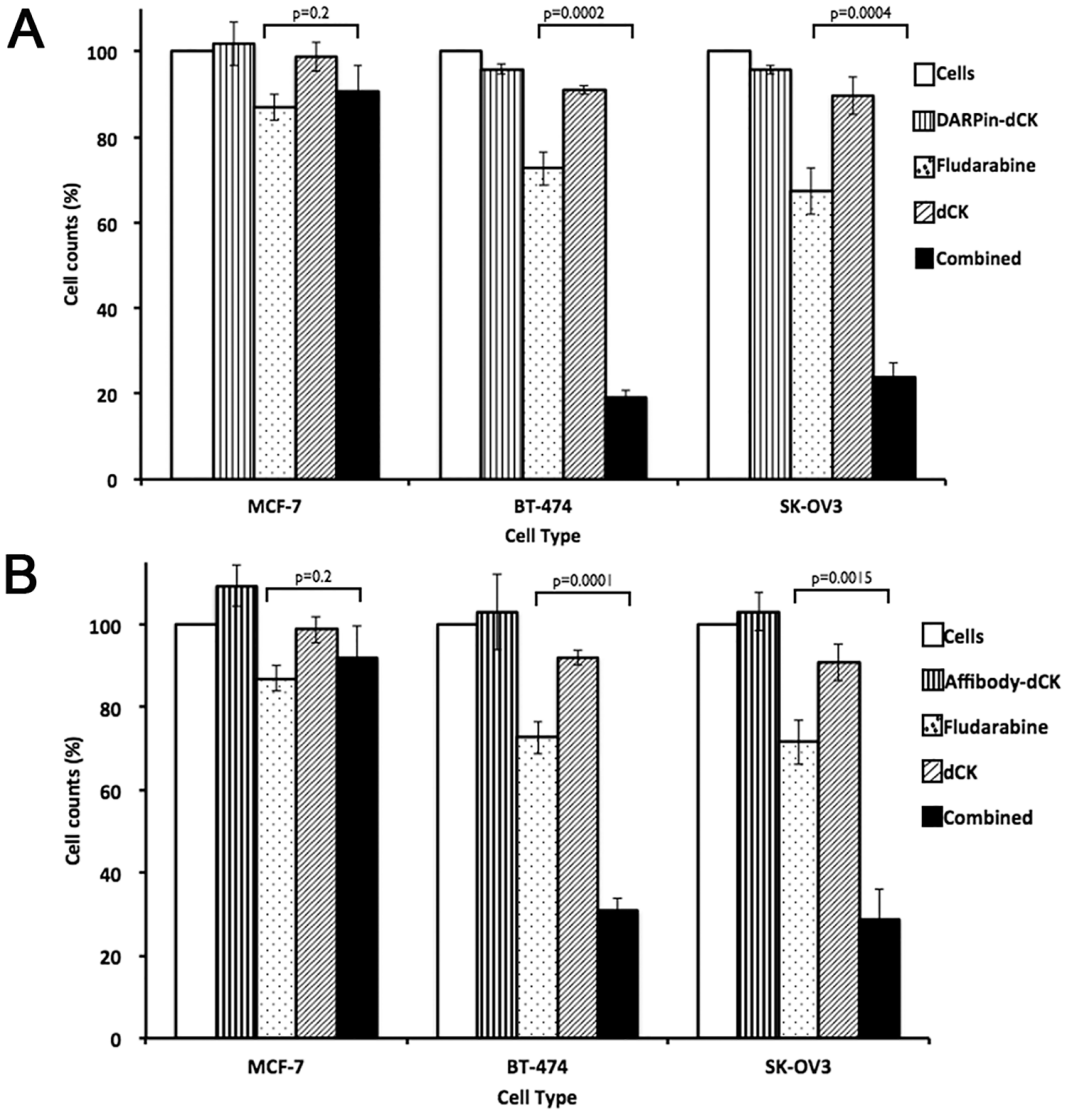
Effect of treatment of bi-modular fusion proteins on proliferation of cancer cells. (A) Seven thousand cells were treated with (i) buffer as control, (ii) bi-modular fusion protein (DARPin-dCK at 5 μM, panel A or (B) Affibody-dCK at 10 μM, panel B), (iii) fludarabine (0.25 μM), (iv) dCK (10 μM) and (v) as combination of fludarabine and bi-modular fusion protein at the same concentrations when tested by themselves. After the addition of the above reagents, cells were further incubated at 37°C for 96 h in 96 well plates, upon which cell proliferation was assessed using the AlamarBlue assay. The cell proliferation signal was normalized to the buffer control signal, which was set at 100%. Results are shown for the Her2-negative MCF-7 and Her2-positive BT-474 and SK-OV3 cell lines. Error bars correspond to standard deviations of triplicate measurements. p values were calculated using the Student’s T-test macros in Microsoft Excel (tail = 1, type = 3).

Treatment with the anti-Her2 modules by themselves (DARPin and affibody), under the experimental conditions tested, had no effect on cell proliferation (Fig 5A & 5B). At the fludarabine dose chosen, cell proliferation is reduced by 10–30%, dependent on cell type, presumably due to endogenous dCK. As expected, addition of dCK by itself has no significant effect on cell proliferation, nor did incubating cells with dCK and fludarabine increase the cell-killing power of the nucleoside analog (S4 Fig). In contrast, the combination of fludarabine with DARPin-dCK and (Fig 5A) affibody-dCK (Fig 5B) shows decreased proliferation of Her2-positive cells (80% or more). Significantly, the combination treatment has no effect on the Her2-negative MCF-7 cells. The enhanced cell-killing ability of the combination therapy is presumably due to the increased activation of fludarabine provided by the intracellularly delivered dCK enzyme.

### Detection sensitivity of proposed treatment method

**Fig 6:**
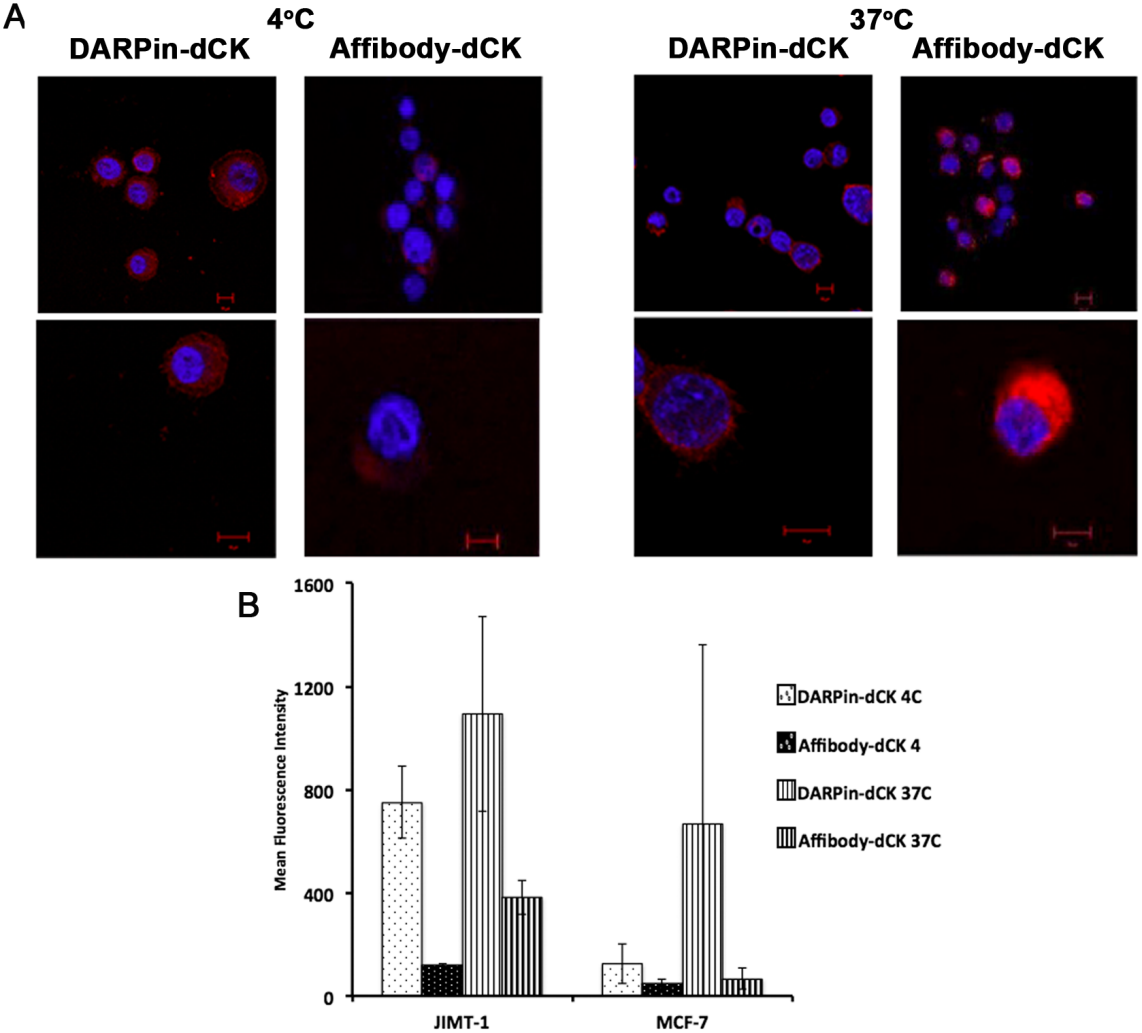
Binding of fusion proteins to low Her2-expressing cancer cells. (A) JIMT-1 cells were treated and imaged as in Fig 4. (B) Flow cytometry assay of reagents binding to Her2 expressing JIMT-1 cells. Cells were treated and counted as in Fig 5A. Mean fluorescence intensity of reagent treated JIMT-1 cells are compared to Her2- MCF-7 cells. Error bars correspond to standard deviations of triplicate measurements.

Herceptin has been used as successful adjuvant therapy for Her2-positive cancers [38]. Herceptin works in part by promoting rapid internalization and degradation of Her2, thereby blocking signaling by the receptor and leading to a loss of cell proliferation [39]. It is also thought to act by triggering antibody-dependent cell-mediated cytotoxicity (ADCC) [40]. However, there are some Her2-positive cancers that are insensitive to Herceptin treatment [41]. The JIMT-1 cell line was generated from one such Herceptin-insensitive tumor [42] and shows enhanced levels of Her2 receptor levels compared to Her2-negative cells, but still lower than other Her2-positive cells (S2B Fig; compare to S2A Fig). As the mode of action of our potential therapy does not act via Her2 signaling, we speculated that our reagents could act on Herceptin-insensitive cells that maintain detectable expression levels of the receptor. A prerequisite of our therapeutic strategy is the binding of the fusion protein to the target cells followed by efficient internalization. Given their low levels of Her2 receptor expression, we tested if JIMT-1 cells are capable of internalizing our scaffold-enzyme fusions. We found that our fusion proteins were able to bind and be internalized by Her2 receptors on JIMT-1 cells (Fig 6A) and that this binding and internalization is significantly greater in Her2-positive (low) JIMT-1 cells than in the Her2-negative MCF-7 cells (Fig 6B). This suggests that our proposed strategy has the potential of distinguishing between low Her2 expressing cancers and Her2-negative cells, thereby suggesting the possibility of a more robust treatment method.

### Summary and prospective

The described anti-cancer approach relies on three factors. One, a cancer cell targeting module. Notably, as additional cancer markers are identified, it becomes possible to develop affinity reagents towards these markers, and hence our approach can conceptually be used to combat diverse cancer types. The second factor is a prodrug-activating enzyme. The engineered dCK variant we used, by its nature of enhanced activity, is well suited as being this factor. Moreover, being a derivative of the human enzyme, it is less likely to be immunogenic. Last, the third factor of this therapeutic approach is the nucleoside analog. Here we tested fludarabine, which seemed to work quite well. However, we realize that the ideal nucleoside analog would not be activated by wild type, endogenous dCK, and only by the engineered version. In this way, endogenous dCK present in healthy tissue will not convert the prodrug to its active cytotoxic form, but the cancer-cell delivered engineered dCK would. This would greatly improve the therapeutic index of the system.

In conclusion, we were successful in generating anti-Her2 module-dCK fusion proteins that are capable of recognizing the cancer biomarker, Her2. These fusion proteins are internalized and by delivering dCK into cells increase the activation potential of dCK-dependent nucleoside analogs (such as fludarabine). The targeting sensitivity of these fusion proteins is such that they are able to distinguish between cancer cells that express low levels of Her2 and Her2-negative cancer cells, thereby suggesting a possibility for a more ubiquitous treatment strategy. This is significant because current treatment methods using nucleoside analog drugs although potent do not inherently distinguish between cancer cell lines and normal cell lines and their anti-cancer activity is attributable to the high proliferation rate of cancer cells compared to normal cells. But with our strategy, we can specifically target the anti-proliferative activity of nucleoside analogs to cancer cells that express the specific biomarker of interest and thus link the therapeutic to the target. This proof or principle can be applied for any biomarker of diseases resulting from abnormal cell proliferation.

## Supporting Information

S1 Fig Amino acid sequence of the DARP-dCK and Affibody-dCK constructs. (DOCX)

S2 Fig Her2 expression in cancer cell lines. (DOCX)

S3 Fig Dose-response for fludarabine. (DOCX)

S4 Fig Effect of dCK and fludarabine treatment on proliferation of cancer cell lines. (DOC)
