# Supplementary material for: Targeted Delivery of Deoxycytidine Kinase to Her2-Positive Cells Enhances the Efficacy of the Nucleoside Analog Fludarabine
Source: PLoS One. 2016 Jun 9;11(6):e0157114. doi: 10.1371/journal.pone.0157114 (PMC4900609; doi:10.1371/journal.pone.0157114)
Supplement: S1 Fig — (DOCX) [file pone.0157114.s001.docx]

**S1 Fig:** **Primary structure of constructs**. The anti-Her2 DARPin (blue) and Affibody (yellow) fused to engineered dCK (purple) are shown. The T7 and c-myc epitope sequences are indicated by red and green lettering, respectively.

DARP-dCK

TYMASMTGGQ QMGTSDLGKK LLEAARAGQD DEVRILMANG ADVNAKDEYG LTPLYLATAH

GHLEIVEVLL KNGADVNAVD AIGFTPLHLA AFIGHLEIAE VLLKHGADVN AQDKFGKTAF

DISIGNGNED LAEILQKLEQ KLISEEDLHM ATPPKRSCPS FSASSEGTRI KKISIEGNIA

AGKSTFVNIL KQLSEDWEVV PEPVARWCNV QSTQDEFEEL TMEQKNGGNV LQMMYEKPER

WSFTFQTYAC LSMIRAQLAS LNGKLKDAEK PVLFFERSVY SARYIFASNL YESESMNETE

WTIYQDWHDW MNNQFGQSLE LDGIIYLQAT PETCLHRIYL RGRNEEQGIP LEYLEKLHYK

HESWLLHRTL KTNFDYLQEV PILTLDVNED FKDKYESLVE KVKEFLSTL

Affibody-dCK

NKEMRNAYWE IALLPNLNNQ QKRAFIRSLY DDPSQSANLL AEAKKLNDAQ APKAAAFEQK

LISEEDLHMA TPPKRSCPSF SASSEGTRIK KISIEGNIAA GKSTFVNILK QLSEDWEVVP

EPVARWCNVQ STQDEFEELT MEQKNGGNVL QMMYEKPERW SFTFQTYACL SMIRAQLASL

NGKLKDAEKP VLFFERSVYS ARYIFASNLY ESESMNETEW TIYQDWHDWM NNQFGQSLEL

DGIIYLQATP ETCLHRIYLR GRNEEQGIPL EYLEKLHYKH ESWLLHRTLK TNFDYLQEVP

ILTLDVNEDF KDKYESLVEK VKEFLSTL

Color legend:

Red: T7 epitope tag (present only in the DARP-dCK construct)

Blue: DARPin

Green: c-myc epitope tag

Purple: dCK

Yellow: affibody

Gray: extra residues due to cloning
