# Supplementary material for: Targeted Delivery of Deoxycytidine Kinase to Her2-Positive Cells Enhances the Efficacy of the Nucleoside Analog Fludarabine
Source: PLoS One. 2016 Jun 9;11(6):e0157114. doi: 10.1371/journal.pone.0157114 (PMC4900609; doi:10.1371/journal.pone.0157114)

**S2 Fig. Her2 expression in cancer cell lines.** The amount of Her-2 receptor expressed in cancer cell lines was measured using the Human Her2 (total) ELISA kit, according to the manufacturer’s protocol. **A.** Copies of Her2/cell for the two Her2 positive cancer cell lines, BT-474 and SK-OV3 are shown compared to the Her2 negative (non-expressing) cancer cell line, MCF-7. **B.** Copies of Her2/cell for the low-expressing JIMT-1 cancer cell line is plotted along with those for the Her2 negative cell line, MCF-7. Error bars correspond to standard deviations of three independent trials for all samples shown.


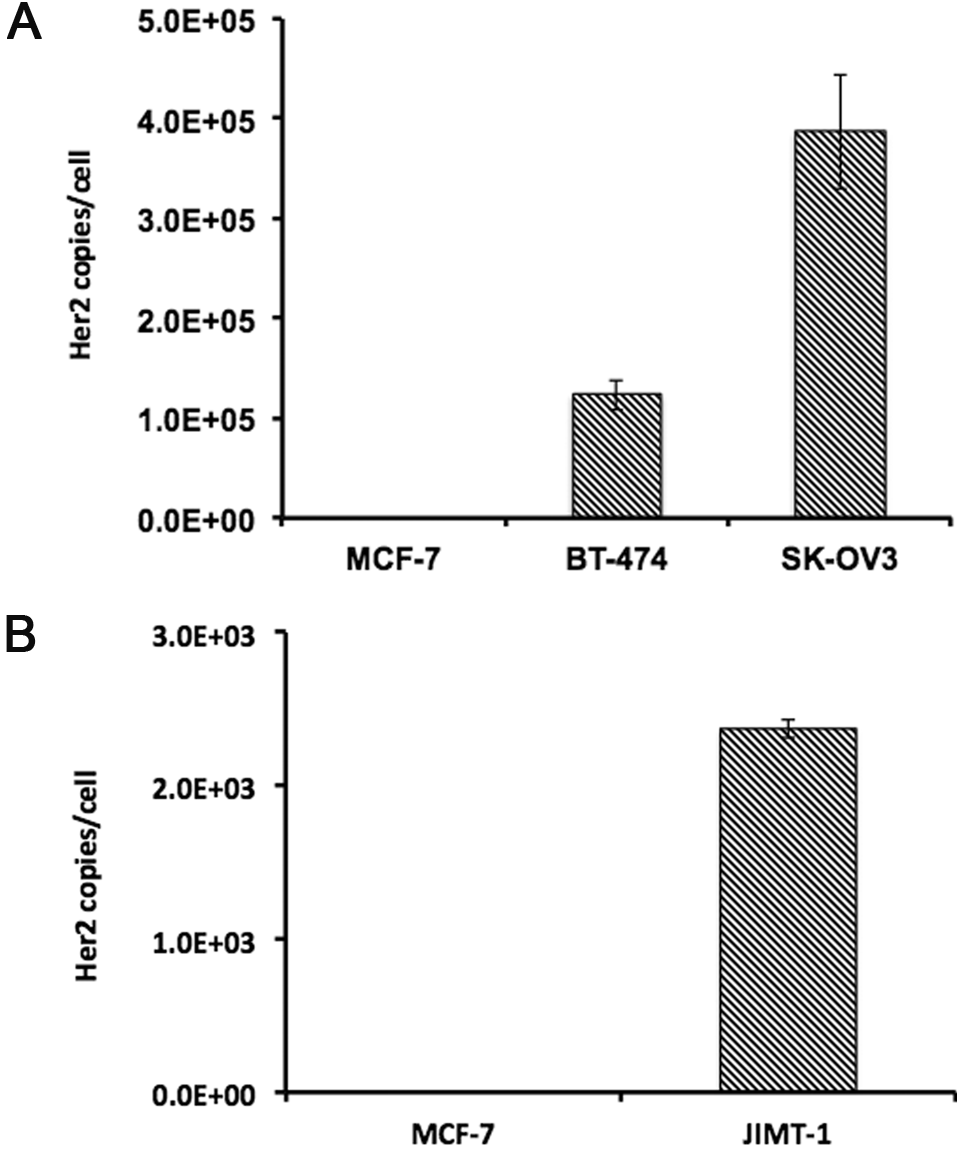

Supplement: S2 Fig — (DOCX) [file pone.0157114.s002.docx]
