# Supplementary material for: Targeted Delivery of Deoxycytidine Kinase to Her2-Positive Cells Enhances the Efficacy of the Nucleoside Analog Fludarabine
Source: PLoS One. 2016 Jun 9;11(6):e0157114. doi: 10.1371/journal.pone.0157114 (PMC4900609; doi:10.1371/journal.pone.0157114)

**S3 Fig. Dose-response for fludarabine.** Cell lines were treated with different concentrations of fludarabine, and the number of live cells was plotted against concentration. One quarter μM (red box) was picked as ideal concentration for cell proliferation assay. Error bars correspond to standard deviation of triplicate measurements.


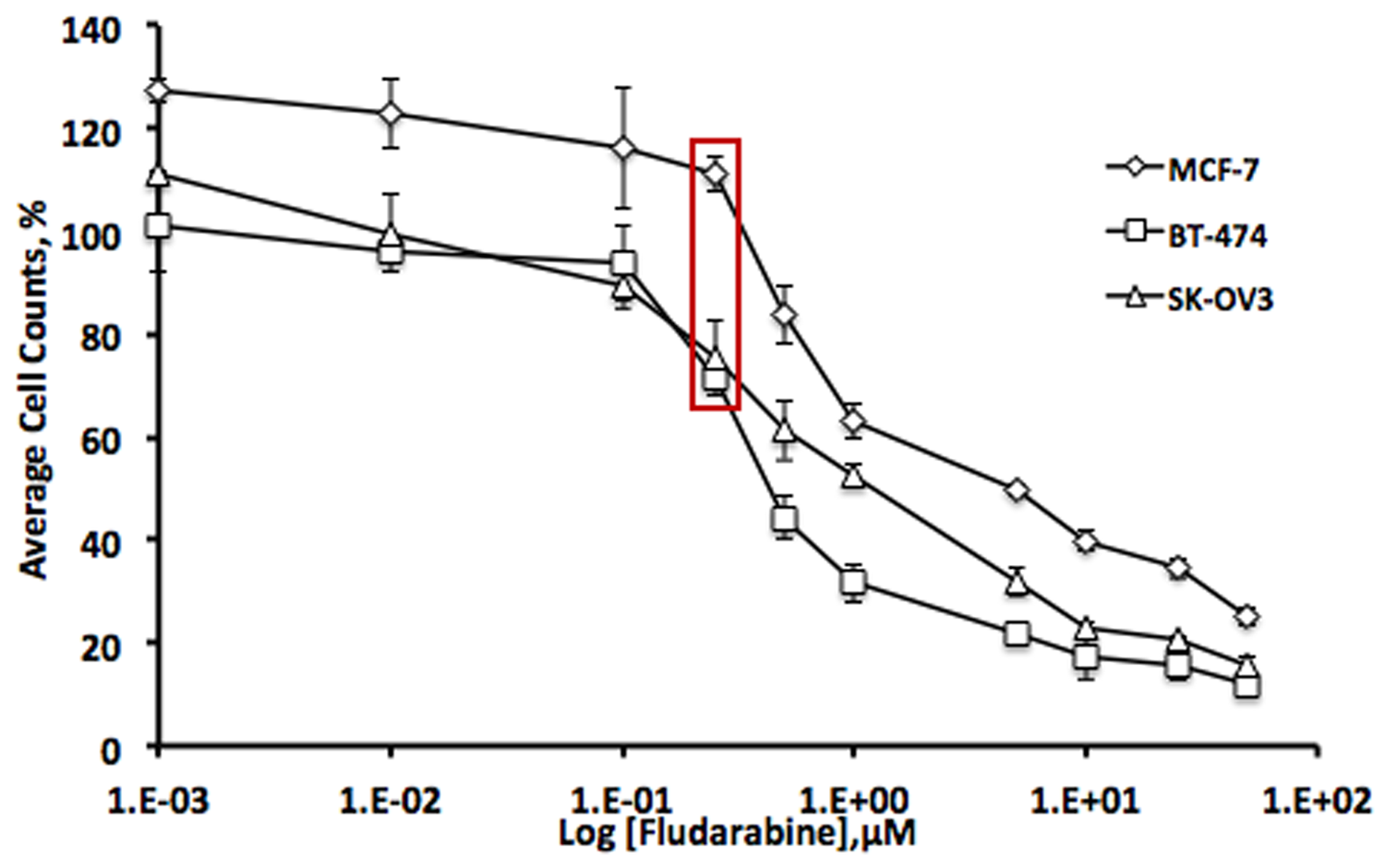

Supplement: S3 Fig — (DOCX) [file pone.0157114.s003.docx]
