# Supplementary material for: Targeted Delivery of Deoxycytidine Kinase to Her2-Positive Cells Enhances the Efficacy of the Nucleoside Analog Fludarabine
Source: PLoS One. 2016 Jun 9;11(6):e0157114. doi: 10.1371/journal.pone.0157114 (PMC4900609; doi:10.1371/journal.pone.0157114)

**S4 Fig. Effect of dCK and fludarabine treatment on proliferation of cancer cell lines.** Cells were treated as in Figure 6B, and the number of proliferating cells was plotted in percentages for each cell type. Error bars correspond to standard deviation of three independent trials.

Figure S4:


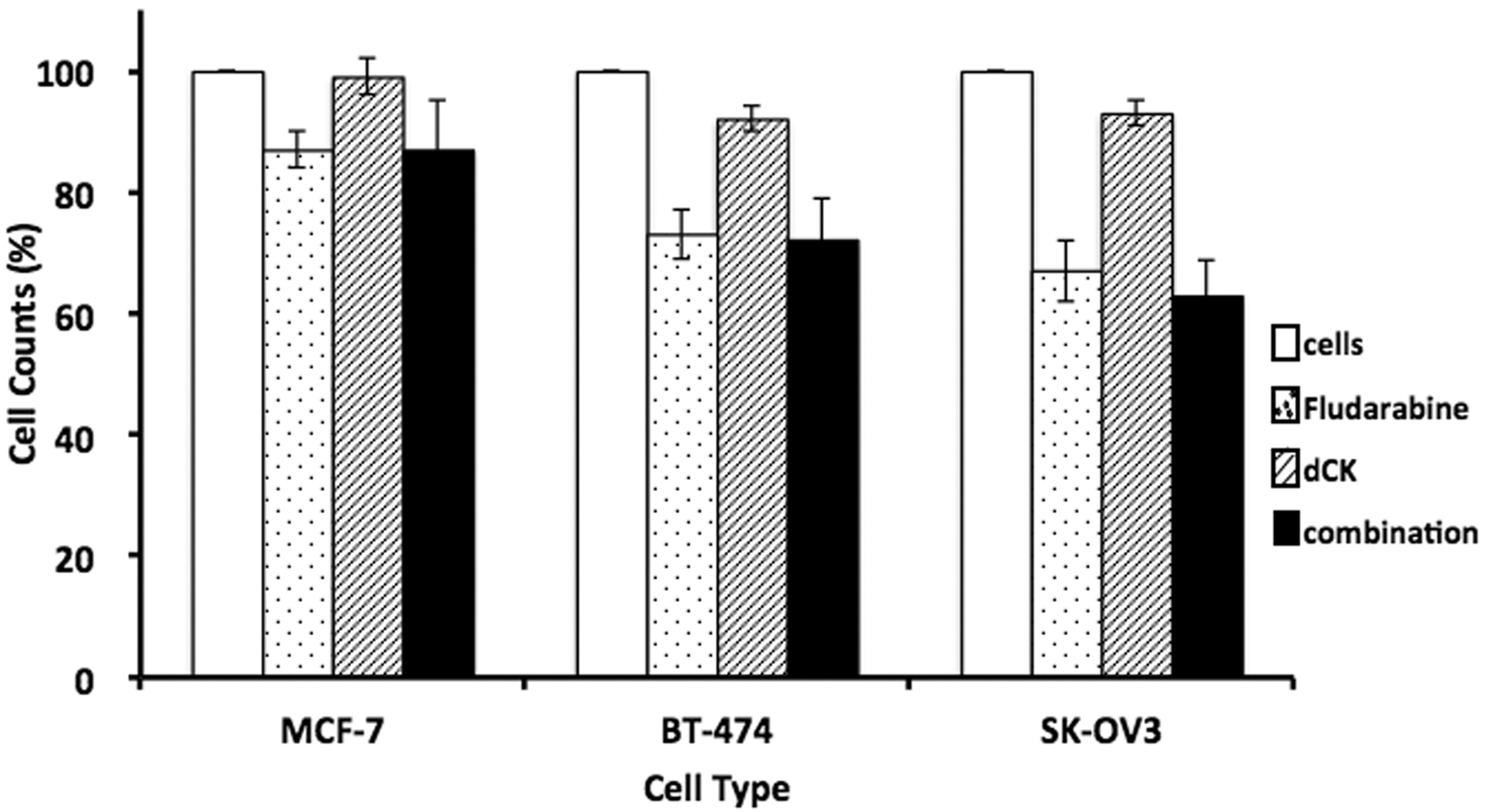

Supplement: S4 Fig — (DOC) [file pone.0157114.s004.doc]
